# Supplementary material for: Patient characteristics and changes in anxiety symptoms in patients with panic disorder: Post-hoc analysis of the PARADIES cluster randomised trial
Source: PLoS One. 2022 Sep 29;17(9):e0275509. doi: 10.1371/journal.pone.0275509 (PMC9521898; doi:10.1371/journal.pone.0275509)
Supplement: S1 File — (PDF) [file pone.0275509.s001.pdf]

**S1:** Pearson correlation coefficient (for continuous variables)/t-test for categorical variables

| | | BAI (T0) | Age: years | Education time: years | Illness duration: months | Depression scale (PHQ-9 T0) | Patient assessment of chronic illness care (PACIC) |
|---|---|---|---|---|---|---|---|
| All | BAI difference | 0.470 (<0.0001) | -0.065 (0.3174) | 0.017 (0.7905) | 0.010 (0.8816) | 0.093 (0.1527) | -0.057 (0.3803) |
| Intervention group | BAI difference | 0.440 (<0.0001) | -0.143 (0.1066) | 0.041 (0.6453) | 0.004 (0.9613) | 0.205 (0.0204) | -0.016 (0.8599) |
| Control group | BAI difference | 0.526 (<0.0001) | -0.016 (0.8684) | 0.005 (0.9552) | 0.010 (0.9175) | -0.026 (0.7865) | -0.075 (0.4429) |

BAI: Beck-Anxiety-Inventory

| All | | BAI difference (mean/SD) | p-value ttest |
|---|---|---|---|
| Sex | Male | 5.0±13.1 | 0.0472 |
| | Female | 9.2±13.8 | |
| Multimorbidity | Yes | 7.7±14.0 | 0.5884 |
| | no | 8.6±13.5 | |
| Benzodiazepine | Yes | 11.6±10.8 | 0.3143 |
| | No | 7.9±13.9 | |
| Antidepressant | Yes | 6.7±14.7 | 0.1379 |
| | No | 9.4±12.8 | |
| Polypharmacy | Yes | 6.6±13.6 | 0.3996 |
| | No | 8.5±13.8 | |
| Psychotropic polypharmacy | Yes | 9.6±12.9 | 0.3939 |
| | No | 7.7±13.9 | |

| Intervention group | | BAI difference (mean/SD) | p-value ttest |
|---|---|---|---|
| Sex | Male | 8.7±11.5 | 0.4345 |
| | Female | 10.5±12.1 | |
| Multimorbidity | Yes | 9.5±11.9 | 0.6014 |
| | no | 10.6±12.1 | |
| Benzodiazepine | Yes | 9.1±8.5 | 0.2027 |
| | No | 10.1±12.3 | |
| Antidepressant | Yes | 9.7±13.7 | 0.8192 |
| | No | 10.3±10.7 | |
| Polypharmacy | Yes | 10.2±12.2 | 0.9406 |
| | No | 10.0±12.0 | |
| Psychotropic polypharmacy | Yes | 9.9±11.7 | 0.8410 |
| | No | 10.5±13.3 | |

| Control group | | BAI difference (mean/SD) | p-value ttest |
|---|---|---|---|
| Sex | Male | -0.5±13.7 | 0.0231 |
| | Female | 7.6±15.4 | |
| Multimorbidity | Yes | 5.6±15.9 | 0.8556 |
| | no | 6.2±14.8 | |
| Benzodiazepine | Yes | 18.5±14.7 | 0.0938 |
| | No | 5.4±15.2 | |
| Antidepressant | Yes | 3.9±15.1 | 0.1576 |
| | No | 8.1±15.5 | |
| Polypharmacy | Yes | 2.8±14.3 | 0.2754 |
| | No | 6.7±15.6 | |
| Psychotropic polypharmacy | Yes | 8.7±12.7 | 0.3246 |
| | No | 5.1±15.9 | |
