# Supplementary material for: Patient characteristics and changes in anxiety symptoms in patients with panic disorder: Post-hoc analysis of the PARADIES cluster randomised trial
Source: PLoS One. 2022 Sep 29;17(9):e0275509. doi: 10.1371/journal.pone.0275509 (PMC9521898; doi:10.1371/journal.pone.0275509)

S2: Regression analysis, R2 and model residuals

Table 3. Findings of univariate and multivariate linear regression models for all study participants (n=236)

	**Univariate regression coefficient (95% CI)**	**Multivariate regression coefficient (optimal model after the stepwise procedure; 95% CI)#**
Allocated to intervention group (vs treatment as usual)	4.2 (0.6 to 7.7) *	5.7 (2.6 to 8.8)***
**Demographics**		
Age [years]	-0.1 (-0.2 to 0.1)	-0.1 (-0.2 to 0.02)
Female sex (vs male)	4.1 (0.1 to 8.2)*	3.3 (-0.2 to 6.7)·
Education time [years]	0.08 (-0.5 to 0.7)	
**Clinical parameters at baseline**		
Anxiety symptom severity (BAI T0)	0.5 (0.4 to 0.7)***	0.8 (0.6 to 1.0) ***
Illness Duration [months]	0.01 (-0.1 to 0.1)	0.2 (0.02-0.4)*
Depression scale (PHQ-9)	0.2 (-0.1 to 0.5)	-0.9 (-1.4 to -0.5)***
Multimorbidity (vs not multimorbid)	-1.0 (-4.5 to 2.6)	
Patient assessment of chronic illness care (PACIC)	-0.3 (-1.0.1 to 0.4)	
**Medication use at baseline**		
Benzodiazepine (yes vs no)	3.7 (-3. to 10.9)	10.4 (-1.3 to 22.0)·
Antidepressant (yes vs no)	-2.7 (-6.2 to 0.9)	
Antidepressant DDD	0.4 (-2.0 to 2.9)	
Polypharmacy (≥5 medicines vs <5)	-1.9 (-6.3 to 2.5)	-3.2 (-7.2 to 0.8)
Psychotropic polypharmacy (≥2 psychotropic medicines vs <2)	1.9 (-2.5 to 6.2)	
**Interactions**		
Allocated treatment *Anxiety symptoms (BAI)at baseline		-0.3 (-0.6 to 0.005)·
Allocated treatment* Depression scale (PHQ-9) at baseline		0.8 (0.2 to 1.5)*
Allocated treatment* Illness duration [months] at baseline		-0.2 (-0.5 to 0.001)·
R^2		0.3105

·<0.1; *<0.05; **<0.01; ***<0.001; # independent variables multivariate model: group, age, sex, education time, baseline anxiety symptom severity, illness duration, depression scale, multimorbidity, patient assessment of chronic illness care, benzodiazepine, antidepressant, polypharmacy, psychotropic polypharmacy

Model	Variablen	R^2
All study participants	Group+PHQ-9+ Group*PHQ-9	0.2792
	Group+PHQ-9+Group*PHQ-9+BAI+ Group*BAI	0.2852
	Group+PHQ-9+ Group*PHQ-9+BAI+Group*BAI+ Illness Duration + Group*Illness Duration	0.2859
	Group +PHQ-9+ Group*PHQ-9+BAI+Group*BAI+ Illness Duration+ Group*Illness Duration+Sex	0.2930
	Group +PHQ-9+ Group*PHQ-9+BAI+Group*BAI+ Illness Duration+ Group*Illness Duration+Sex+Age	0.3021
	Group +PHQ-9+ Group*PHQ-9+BAI+Group*BAI+ Illness Duration+ Group*Illness Duration+Sex+Age+ Polypharmacy	0.3072
	Group +PHQ-9+ Group*PHQ-9+BAI+Group*BAI+	0.3105

	Illness Duration+ Group*Illness Duration+Sex+Age+Polypharmacy+ Benzodiazepine+Group*Benzodiazepine

Residual analysis	

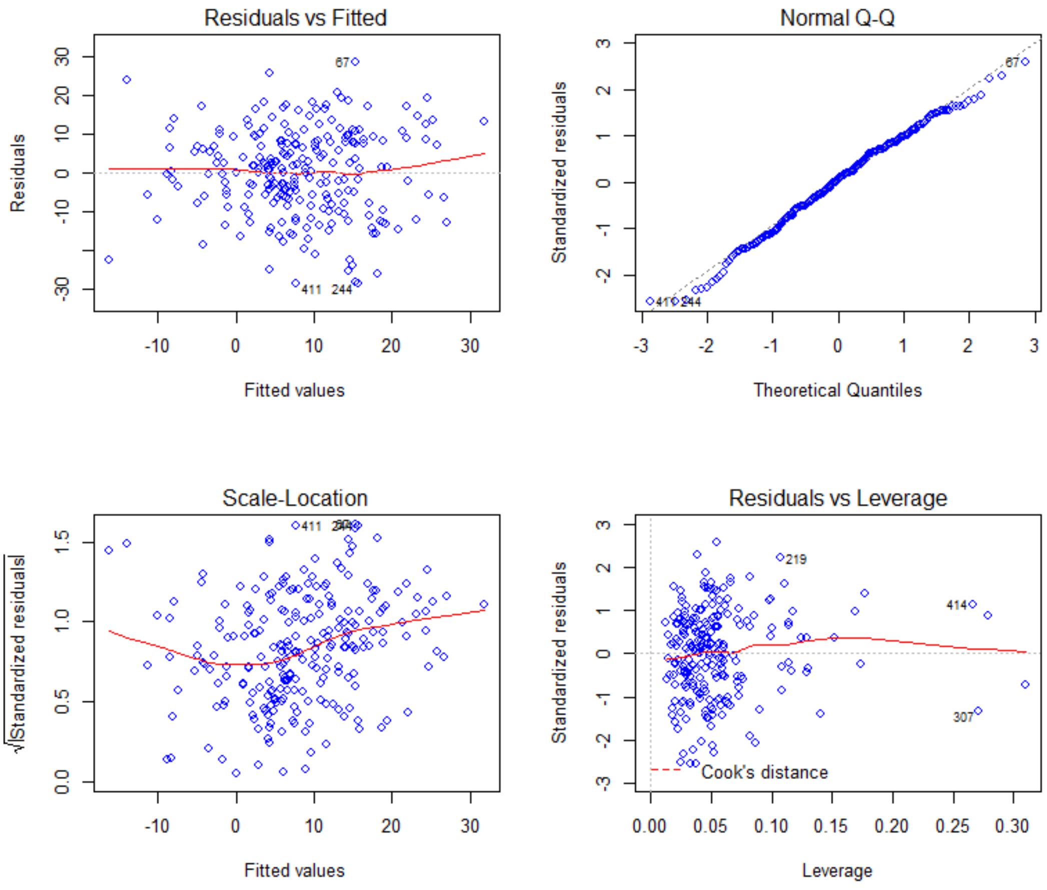

Table 4: Findings of regression analyses stratified by intervention and control status.

	Control group		Intervention group	
	Univariate regression coefficient (95% CI)	Multivariate regression coefficient (optimal model after stepwise procedure; 95% CI)#	Univariate regression coefficient (95% CI)	Multivariate regression coefficient (optimal model after stepwise procedure; 95% CI)#
**Demographics**				
Age [years]	-0.02 (-0.2 to 0.2)		-0.1 (-0.3 to 0.0)	-0.2 (-0.3 to -0.02)*
Female sex (vs male)	8.1 (1.1 to 15.1)*	4.5 (-1.3 to 10.3)	1.9 (-2.8 to 6.6)	
Education time [years]	0.03 (-1.0 to 1.0)		0.2 (-0.5 to 0.9)	
**Clinical parameters at baseline**				
Anxiety symptom severity (BAI T0)	0.6 (0.4 to 0.8)***	0.8 (0.6 to 1.0) ***	0.4 (0.3 to 0.6)***	0.5 (0.3 to 0.6) ***
Illness Duration [months]	0.01 (-0.2 to 0.2)	0.2 (-0.03 to 0.4)·	0.0 (-0.1 to 0.2)	
Depression scale (PHQ-9)	-0.1 (-0.6 to 0.5)	-0.9 (-1.4 to -0.4)***	0.4 (0.1 to 0.8)*	
Multimorbidity (vs not multimorbid)	-0.5 (-6.4 to 5.4)		-1.1 (-5.3 to 3.1)	
Patient assessment of chronic illness care (PACIC)	-0.4 (-1.5 to 0.7)		-0.1 (-0.9 to 0.8)	
**Medication use at baseline**				
Benzodiazepine (yes vs no)	13.1 (-2.3 to 28.5)	9.6 (-2.8 to 21.9)	-1.0 (-8.5 to 6.5)	
Antidepressant (yes vs no)	-4.2 (-10.0 to 1.6)		-0.5 (-4.8 to 3.8)	
Antidepressant DDD	-0.1 (-4.8 to 4.6)		0.7 (-1.9 to 3.4)	
Polypharmacy (≥5 medicines vs <5)	-3.9 (-11.1 to 3.2)	-6.0 (-11.8 to -0.2)*	0.2 (-5.1 to 5.5)	
Psychotropic polypharmacy (≥2 psychotropic medicines vs <2)	3.6 (-3.6 to 10.7)		0.5 (-4.7 to 5.8)	
**Delivery modalities**				
Appointment 4 performed	Not applicable		4.7 (-0.4 to 9.9)	
Telephone contacts	Not applicable		1.0 (-0.04 to 1.9)	
Additional contacts	Not applicable			
0			Reference	
1			-3.2 (-0.5 to 3.1)	
2			4.6 (-9.2 to 18.3)	
R^2		0.3759		0.2117

·<0.1; *<0.05; **<0.01; ***<0.001; # independent variables multivariate model: age, sex, education time, baseline anxiety symptom severity, illness duration, depression scale, multimorbidity, patient assessment of chronic illness care, benzodiazepine, antidepressant, polypharmacy, psychotropic polypharmacy

Model	Variablen	R^2
Intervention group	BAI	0.1872
	BAI+Age	0.2117
Control group	BAI	0.2700
	BAI+PHQ-9	0.3341
	BAI+PHQ-9+Polypharmacy	0.3485
	BAI+PHQ-9+Polypharmacy +Illness Duration	0.3601
	BAI+PHQ-9+Polypharmacy +Illness Duration+Benzodiazepine	0.3679
	BAI+PHQ-9+Polypharmacy+Illness Duration+Benzodiazepine+Sex	0.3759

Group: intervention or control; PHQ-9: Depression scale at baseline; BAI: Anxiety symptom severity at baseline;

Control group: Residual analysis – multivariate model

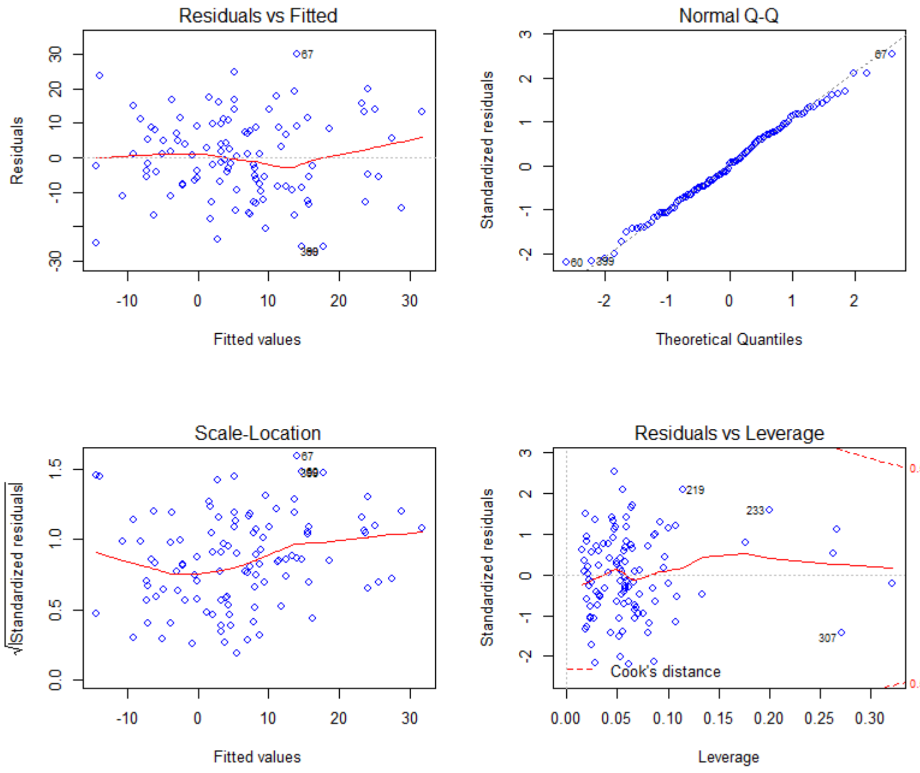

Intervention group: Residual analysis – multivariate model

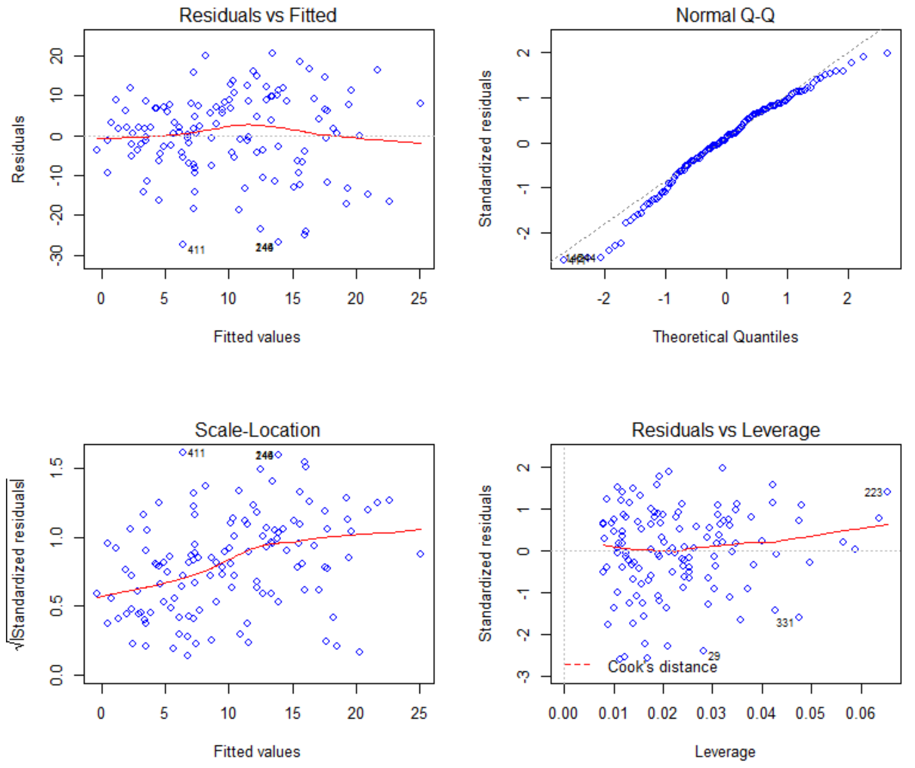

Table 5. Findings of regression analyses stratified by use vs non-use of antidepressants and/or benzodiazepines.

	**Non-users (n=120) of antidepressants or benzodiazepines**		**Users (n=116) of antidepressants or benzodiazepines**	
	Univariate regression coefficient (95% CI)	Multivariate regression coefficient (optimal model after the stepwise procedure; 95% CI)#	Univariate regression coefficient (95% CI)	Multivariate regression coefficient (optimal model after the stepwise procedure; 95% CI)#
Allocated to intervention group (vs treatment as usual)	3.0 (-1.8 to 7.7)	4.8 (0.8 to 8.8)*	5.0 (-0.2 to 10.3)	5.8 (1.4 to 10.1)**
**Demographics**				
Age [years]	-0.1 (-0.2 to 0.1)		-0.1 (-0.3 to 0.1)·	
Female sex (vs male)	-0.5 (-6.0 to 5.1)		8.4 (2.5 to 14.3)**	5.5 (0.4 to 10.6)*
Education time [years]	0.2 (-0.7 to 1.1)	0.8 (0.0 to 1.5)*	-0.02 (-0.8 to 0.8)	
**Clinical parameters at baseline**				
Anxiety symptom severity (BAI T0)	0.6 (0.4 to 0.7)***	0.6 (0.4 to 0.8) ***	0.5 (0.3 to 0.7)***	0.7 (0.5 to 0.9) ***
Illness Duration [months]	0.01 (-0.2 to 0.2)		0.01 (-0.2 to 0.2)	
Depression scale (PHQ-9)	0.7 (0.3 to 1.1)**		-0.1 (-0.6 to 0.3)	-0.6 (-1.1 to -0.2)**
Antidepressant DDD	4.2 (0.2 to 8.1)*			
Multimorbidity (vs not multimorbid)	-1.0 (-5.7 to 3.7)		-0.8 (-6.2 to 4.5)	
Patient assessment of chronic illness care (PACIC)	-0.7 (-1.6 to 0.3)		0.1 (-0.9 to 1.1)	
**Medication use at baseline**				
Polypharmacy (≥5 medicines vs <5)	3.5 (-2.9 to 10.0)·	4.4 (-1.0 to 9.9)·	-5.4 (-11.5 to 0.7)·	-7.1 (-12.1 to -2.1)**
Psychotropic polypharmacy (≥2 psychotropic medicines vs <2)	5.1 (-5.7 to 15.8)		3.1 (-2.4 to 8.6)	
R^2		0.3039		0.3429

·<0.1; *<0.05; **<0.01; ***<0.001; # independent variables multivariate model: group, age, sex, education time, baseline anxiety symptom severity, illness duration, depression scale, multimorbidity, patient assessment of chronic illness care, benzodiazepine, antidepressant, polypharmacy, psychotropic polypharmacy

Model	Variablen	R^2
Users of antidepressants and/or benzodiazepines	BAI	0.1841
	BAI+PHQ-9	0.2435
	BAI+PHQ-9+Polypharmacy	0.2825
	BAI+PHQ-9+Polypharmacy+Group	0.3215
	BAI+PHQ-9+Polypharmacy+Group+Sex	0.3429
Non-users of antidepressants and benzodiazepines	BAI	0.2619
	BAI+Group	0.2823
	BAI+Group+Education time	0.2943
	BAI+Group+Education time+Polypharmacy	0.3039

Group: intervention or control; PHQ-9: Depression scale at baseline; BAI: Anxiety symptom severity at baseline;

Non-users of benzodiazepines or antidepressants (n=120)

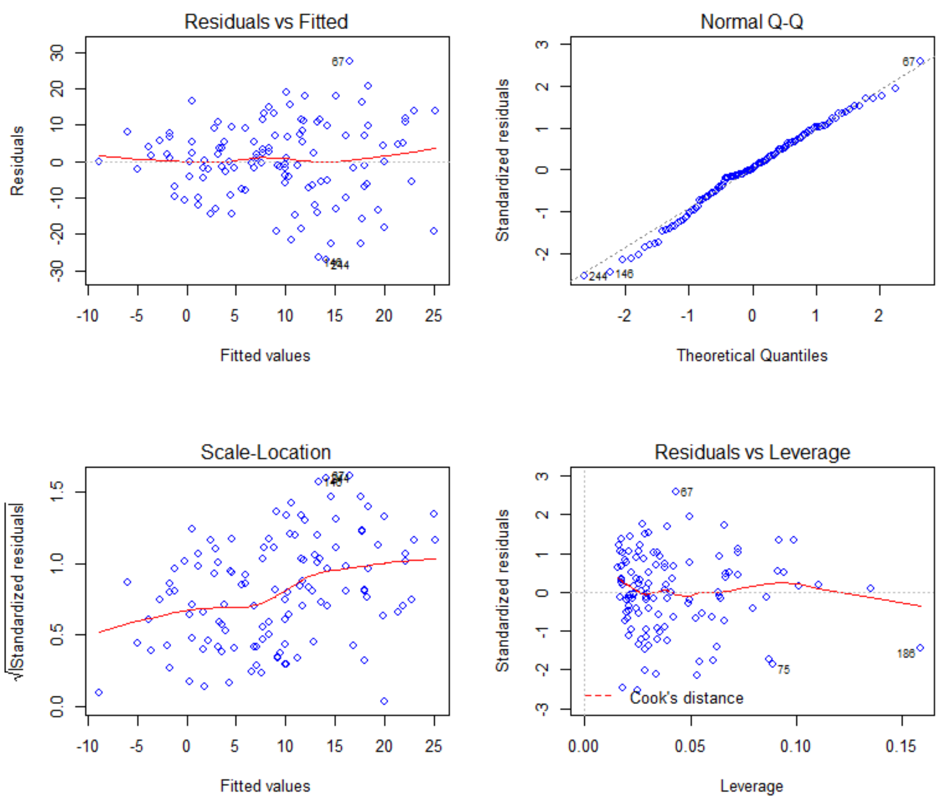

Users of benzodiazepines or antidepressants (n=116)

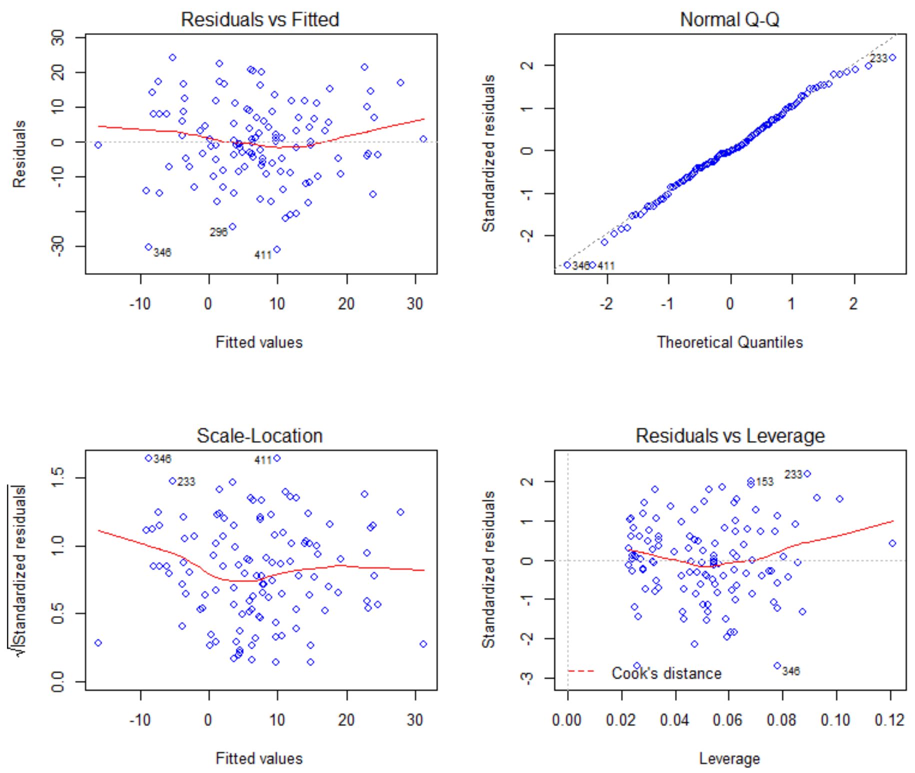

Supplement: S2 File — (PDF) [file pone.0275509.s002.pdf]
