# Supplementary material for: Patient characteristics and changes in anxiety symptoms in patients with panic disorder: Post-hoc analysis of the PARADIES cluster randomised trial
Source: PLoS One. 2022 Sep 29;17(9):e0275509. doi: 10.1371/journal.pone.0275509 (PMC9521898; doi:10.1371/journal.pone.0275509)
Supplement: Attachment — Submitted filename: PONE_D_22_02155_Respons to reviewers.docx [file pone.0275509.s003.docx]

**PONE-D-22-02155**

**Patient characteristics and changes in anxiety symptoms in patients with panic disorder: Post-hoc analysis of the PARADIES cluster randomised trial**

**Response to reviewers**

1) Please ensure that your manuscript meets PLOS ONE's style requirements, including those for file naming.

**OUR RESPONSE:** The manuscript now meets PLOS ONE's style requirements, including those for file naming.

2) We note that you have indicated that data from this study are available upon request. PLOS only allows data to be available upon request if there are legal or ethical restrictions on sharing data publicly. In your revised cover letter, please address the following prompts:
a. If there are ethical or legal restrictions on sharing a de-identified data set, please explain them in detail (e.g., data contain potentially identifying or sensitive patient information) and who has imposed them (e.g., an ethics committee). Please also provide contact information for a data access committee, ethics committee, or other institutional body to which data requests may be sent.
b. If there are no restrictions, please upload the minimal anonymized data set necessary to replicate your study findings as either Supporting Information files or to a stable, public repository and provide us with the relevant URLs, DOIs, or accession numbers. Please see http://www.bmj.com/content/340/bmj.c181.long for guidelines on how to de-identify and prepare clinical data for publication. For a list of acceptable repositories, please see http://journals.plos.org/plosone/s/data-availability#loc-recommended-repositories.
c. We will update your Data Availability statement on your behalf to reflect the information you provide.

**OUR RESPONSE**: The data that support the findings of this study are available from the PARADIES study consortium but restrictions apply to the availability of these data, which were used under license for the current study, and so are not publicly available. Data are however available from the authors upon reasonable request. Data requests may be directed at Jochen.Gensichen@med.uni-muenchen.de

3) We note that you have included the phrase “data not shown” in your manuscript. Unfortunately, this does not meet our data sharing requirements. PLOS does not permit references to inaccessible data. We require that authors provide all relevant data within the paper, Supporting Information files, or in an acceptable, public repository. Please add a citation to support this phrase or upload the data that corresponds with these findings to a stable repository (such as Figshare or Dryad) and provide and URLs, DOIs, or accession numbers that may be used to access these data. Or, if the data are not a core part of the research being presented in your study, we ask that you remove the phrase that refers to these data.

**OUR RESPONSE:** We now provide this data (univariate association of treatment modalities with BAI changes) in the new table 4.

4) One of the noted authors is a group or consortium (PARADIES study group). In addition to naming the author group, please list the individual authors and affiliations within this group in the acknowledgments section of your manuscript. Please also indicate clearly a lead author for this group along with a contact email address.

**OUR RESPONSE:** We provide the names of the PARADIES study group at the end of the manuscript

**Reviewer #1:**

1) This is a secondary regression analysis from an already-completed cluster randomized clinical trial. As such, it is an exploratory study, not powered to detect significant treatment differences, and it should be so-stated in the text. The comment "p<.05 is considered significant" means nothing with no adjustment for many, many hypothesis tests. I suggest a comment that "p-values should be interpreted as a guide, rather than as a determinant of significance". Alternatively, if there are confirmatory hypotheses, these should be stated clearly, power analysis given, and Bonferroni adjustments for multiple tests.

**OUR RESPONSE:** We agree with the reviewer. We have now added in a number of places that we conducted an exploratory analysis and now state in the discussion under limitations:

*“This is a post-hoc analysis of data from the completed PARADIEs trial and as such it is an exploratory study, which is vulnerable to multiple testing bias. While our findings confirm the findings of the fully powered primary analysis, the identified associations between independent and dependent variables should be interpreted as hypothesis generating rather than hypothesis testing.”*

2) I appreciate that they are concerned about analyzing this data set incorrectly, given they are ignoring the cluster effect, and they do indicate some homogeneity across clusters.

**OUR RESPONSE:** We agree, an analysis with a multi-level model would be the best method. However, due to the lower number of cases in our analysis (n=236) compared to the main study (n=419), only 1-2 patients remain in many centres. Therefore, an analysis with a multi-level model is no longer possible because the models do not converge. Due to the low intraclass correlation coefficient (0.017) in the main study and the associated low cluster effect, we decided to perform the analysis without multi-level models. We now further explain under limitations that *our multi level models did not converge.*

3) Finally, any regression course would require all students to do diagnostics for the model, including residual analyses and graphical inspection. I would expect no less in a scientific paper!

**OUR RESPONSE:** We performed diagnostics for all models including the residual plots. We have added the following sentence in the methods section "Residual plots (residuals vs. fitted values, Q-Q plot, scale-location plot, and residuals vs leverage values) were used to check the assumptions of the linear regression models". In addition, we added the residual plots for the multivariate models in the supporting file 2.

**Reviewer #2:**

1) A large part of the introduction describes the PARADIES study, including the independent variables investigated in the study and their relationship with panic disorder in the introduction section.

**OUR RESPONSE:** We have now added to the introduction the following paragraph to explain the rationale for the independent variables investigated: *In particular, we investigated as influencing factors patient demographics (since age, sex and level of education may influence motivation and/or ability to engage with the intervention (19)), symptoms of other psychiatric co-morbidities and multimorbidity as well as the type and/or number of psychotropic and other drugs used to treat these (since all of these factors may influence the prognosis of panic disorders (20).*

2) Out of 236 patients in the study, 128 were in the intervention group and 108 were in the control group. In the method section, it is explained in detail how the patients were assigned to the groups.

**OUR RESPONSE:** We have now included the following details describing the randomisation process in lines 78 ff: *As described in more detail elsewhere (17), practices first recruited patient participants over a three months time period and were subsequently randomised to provide care as usual or deliver the PARADIES intervention to participating patients under their care (allocation ratio 1:1).*

3) Comparison of both groups (intervention & control) in terms of sociodemographic characteristics (with t test and Chi-square tests for independent groups) and giving p values in Table 1 in the Results section.

**OUR RESPONSE:** We now provide the findings of t test and Chi-square tests and provide p values in Table 1 in the results section.

4) Half of the patients are receiving antidepressant treatment at the beginning, comparing the drug doses used by the patients using antidepressants in terms of equivalence (For example, 10 mg. Escitalopram = 20 mg. Citalopram = 20 mg. Fluoxetine = 50 mg. Sertalin = 20 mg. Paroxetine).

**OUR RESPONSE:** For antidepressant users at baseline, we have calculated the defined daily dose of antidepressants taken and compared them between groups. The data is presented in table 1. We have also included this variable in linear regression analyses and provide the findings in tables 3 to 5.

5) Of the 128 patients, 44 improved, 22 worsened. Of the 106 patients, 44 improved and 39 worsened. Rates are given on worsening only. Giving rates over recovery. (The rate of 74.8% on page 9 should be 64.8%.)

**OUR RESPONSE:** We think there is a confusion between patients and changes in BAI points, which we hope to clarify by the following rewording: *In the intervention group, changes in BAI scores ranged from an improvement by 44 points to a worsening by 22 points, and in the control group from an improvement of 44 points to a worsening of 39 points.* We additionally provide the numbers (%) of patients who improved (had changes to the better) and those who had no changes or had changes to the worse.

6) Adding the following table to the results section and making its statistical analysis.
   The patients in both the intervention group and the control group were divided into groups according to the scores they got from the Beck Anxiety Inventory at the baseline and at the end of the 12-month follow-up, and comparing the two groups.
   0-7 points (No anxiety)
   8-15 points (Mild anxiety)
   16-25 points (Moderate anxiety)
   ≥ 26 points (Severe anxiety)

**OUR RESPONSE:** We agree that the manuscript benefits from this additional analysis. We have added the findings in a new table 2 and summarised them in text as follows: *Table 2 compares the proportions of intervention and control group patients by BAI severity category at baseline and at the end of the study period. While there were no significant differences between intervention and control groups at baseline, the groups differed significantly at 12 months follow up. Over the study period, the proportions of patients with severe symptoms decreased from 51.9% to 38.9% in the control group and more than halved (from 52.3% to 22.7%) in the intervention group, while the proportions of patients with no or minimal symptoms increased from 3.7% to 16.7% in the control group and from 1.6% to 28.1% in the intervention group.*

7) Showing the correlation analysis with a table in the results section in terms of the relationship between the independent variables included in the regression analysis and the dependent variable (Pearson correlation coefficients).

**OUR RESPONSE:** We provide the findings of the correlation analysis in a supporting file 1. We suggest that the findings do not substantially add to the univariate relationships between independent and dependent variables reported in the manuscript and are therefore dispensable in the main manuscript.

8) R2 (R square) changes should be given in multiple regression analysis. It shows how much of the variation in the variance (indicates how much the established model determines the investigated relationship) of the relevant independent variable explains. In this study, the model determined 31.1% of the variance. The share of each independent variable in this percentage should be shown by giving the change in R2 (R square).

**OUR RESPONSE:** In order to find the multivariate model that best explains the change in BAI, we used a stepwise procedure based on the Akaike Information Criteria (AIC). The AIC is similar to the adjusted R squared measure and penalizes when more variables are included in the model. The result of this procedure is a simple model with the optimal number of variables. If present, multicollinearity is also removed by this procedure.

We provide R2 changes in the supporting file 2.

9) Table 2 and Table 3 need to be redone. Multiple regression analyzes should be performed separately for the intervention group and the control group, and the discussion section should be rewritten according to the results of this analysis (Univariate regression analysis does not need to be specified).

**OUR RESPONSE:** We have carefully considered this comment and would like to propose that we keep the analysis for the study population as a whole (where we adjust for allocation status), but additionally provide the findings of analysis stratified by intervention and control group. This approach maximises power for examining explanatory variables and avoids missing differential effects in intervention and control groups. We have applied this approach in the revised manuscript and made amendments to methods, results and discussion accordingly.

10) One of the dilemmas of the study is that BDZ (Benzodiazepine) and AD (Antidepressant) treatments used in baseline affect both baseline anxiety severity and depression severity. Therefore, the effect of the intervention applied in the study will be understood more accurately in a sample that does not use drugs. It is recommended to exclude the patients using drugs in both the 128-person intervention group and the 106-person control group, and to analyze the symptoms above for the new intervention and control groups consisting of patients who do not use drugs, and to interpret the similarities and differences between the results found with the 128 and 106-person samples in the discussion section.

**OUR RESPONSE:** We have further stratified the analysis by use of antidepressants or benzodiazepines and provide the findings in the manuscript and made amendments to methods, results and discussion accordingly. We have opted for this approach of adjusting for allocation status rather than a four way stratification by both intervention and control groups because this would have left few individuals in each stratum, which would have limited power even further.
