# Supplementary material for: Patient characteristics and changes in anxiety symptoms in patients with panic disorder: Post-hoc analysis of the PARADIES cluster randomised trial
Source: PLoS One. 2022 Sep 29;17(9):e0275509. doi: 10.1371/journal.pone.0275509 (PMC9521898; doi:10.1371/journal.pone.0275509)
Supplement: Attachment — Submitted filename: PONE_R2_answers to reviewer.docx [file pone.0275509.s004.docx]

PONE-D-22-02155R1

Patient characteristics and changes in anxiety symptoms in patients with panic disorder: Post-hoc analysis of the PARADIES cluster randomised trial

PLOS ONE

1) The article has some structural problems and this situation continues despite the revision. The independent variables researched in the introduction part are not included enough. Some parts that should be included in the method are included in the introduction.

**Our response:** This study is in an exploratory post hoc analyses of data from the PARDIES trial, and the findings of the primary analysis have been published previously. In our opinion, describing the PARADIES intervention and findings of the trial is essential background information for the reader to understand what the objectives of *this* paper are. We believe the aim of the analysis is clearly stated, namely “to examine variability in the course of anxiety symptom severity at 12 months follow up and to explore patient characteristics, which may explain that variability.”

With regards to describing the independent variables and its rationale in the introduction, we have added additional information in our previous revision upon the reviewer’s request and have made further specifications in the revised manuscript as follows: “In particular, we investigated as explanatory factors patient demographics (since age, sex and level of education may influence motivation and/or ability to engage with the intervention)[19], anxiety symptom severity and illness duration at baseline, co-morbid depression, multimorbidity and polypharmacy as well as the type and/or number of psychotropic drugs used to treat these (since all of these factors may influence the prognosis of panic disorders) [20].”

We suggest that providing this level of detail in the introduction should be sufficient for the reader to appreciate the rationale for our selection of independent variables. We provide further details on these variables and how they were defined in the methods section.

2a) It is a serious problem that the study did not include only panic disorder patients.

**Our response**: We would like to clarify that all included patients had panic disorder. We agree that in patients with comorbid depression and panic disorder, it is more difficult to isolate effects on either condition.

However, anxiety and depression share clinical symptoms and causes due to genetic pleiotropy and share psychological, social, and neurobiological risk mechanisms. As a result, comorbidity of anxiety and depression is the rule rather than an exception. For instance, of the individuals with a primary depression diagnosis in the Netherlands Study of Depression and Anxiety (NESDA), 67% had a current and 75% had a lifetime comorbid anxiety disorder diagnosis. Similarly, of those with a primary anxiety disorder diagnosis, 63% had a current and 81% a lifetime depressive disorder diagnosis. Thus, limiting the trial population to those with anxiety disorder but without symptoms of depression would have substantially compromised the applicability/external validity of the findings.

The PARADIES trial has collected data on both depression and anxiety symptoms and all multivariate analyses in this paper took baseline depression symptom severity into account. Our findings suggest that depression symptom severity may mark a group of patients with a less favourable prognosis (as the analysis of control group patients shows) who may nevertheless be at least partially responsive to the PARADIES intervention (as our analysis of intervention group patient shows).

- Choi, K. W., Kim, Y. K., & Jeon, H. J. (2020). Comorbid anxiety and depression: clinical and conceptual consideration and transdiagnostic treatment. Anxiety Disorders, 219-235.
- Demyttenaere, K., & Heirman, E. (2020). The blurred line between anxiety and depression: hesitations on comorbidity, thresholds and hierarchy. International Review of Psychiatry, 32(5-6), 455-465.
- Groen, R.N., Ryan, O., Wigman, J.T., Riese, H., Penninx, B.W., Giltay, E.J., ... & Hartman, C.A. (2020). Comorbidity between depression and anxiety: assessing the role of bridge mental states in dynamic psychological networks. BMC medicine, 18(1), 1-17.
- Hirschfeld R.M.A. The comorbidity of major depression and anxiety disorders: recognition and management in primary care. Prim Care Companion J Clin Psychiatry. 2001;3(6):244–54.
- Lamers F., van Oppen P., Comijs H.C., Smit J.H., Spinhoven P., Van Balkom A.J.L.M., et al. Comorbidity patterns of anxiety and depressive disorders in a large cohort study: the Netherlands Study of Depression and Anxiety (NESD A). J Clin Psychiatry. 2011;72(3):341–8.
- Ter Meulen, W. G., Draisma, S., van Hemert, A. M., Schoevers, R. A., Kupka, R. W., Beekman, A. T., & Penninx, B. W. (2021). Depressive and anxiety disorders in concert–A synthesis of findings on comorbidity in the NESDA study. Journal of affective disorders, 284, 85-97
- Penninx, B., Pine, D., Holmes, Reif, A. Anxiety disorders, Lancet. 2021 March 06; 397(10277): 914–927.

2b) In addition, the fact that both groups were treated with medication is a situation that may seriously affect the results of the study, and the daily dose of antidepressant parameter added to the table by the researchers does not relieve these reservations.

**Our response**: We agree that including patients with and without anti-anxiety medication introduces heterogeneity in the study population. However, we adjusted for medication use (including dose as requested by the reviewer in his/her last review) at baseline in all analyses. In order to minimise any residual confounding, the reviewer recommended in his/her last review “to exclude the patients using drugs in both the 128-person intervention group and the 106-person control group, and to analyse the symptoms above for the new intervention and control groups consisting of patients who do not use drugs, and to interpret the similarities and differences between the results found with the 128 and 106-person samples in the discussion section.” In our previous revision, we therefore further stratified the analysis by use of antidepressants or benzodiazepines and provide and discuss the findings in the manuscript.

In the current version of the manuscript, we have added the following sentence to the strengths and limitations section in order to address further concerns regarding the medication: “Finally, although the intervention focussed on non-pharmacological treatment options, we cannot exclude that differential use of anti-anxiety medication in intervention and control groups during follow up may have contributed to the observed effects.”

- Gensichen, J., Hiller, T. S., Breitbart, J., Brettschneider, C., Teismann, T., Schumacher, U., Lukaschek, K., Schelle, M., Schneider, N., Sommer, M., Wensing, M., König, H. H., Margraf, J., & Jena-PARADISE Study Group (2019). Panic Disorder in Primary Care. Deutsches Arzteblatt international, 116(10), 159–166. <https://doi.org/10.3238/arztebl.2019.0159>
- Gensichen, J., Hiller, T. S., Breitbart, J., Teismann, T., Brettschneider, C., Schumacher, U., Piwtorak, A., König, H. H., Hoyer, H., Schneider, N., Schelle, M., Blank, W., Thiel, P., Wensing, M., & Margraf, J. (2014). Evaluation of a practice team-supported exposure training for patients with panic disorder with or without agoraphobia in primary care - study protocol of a cluster randomised controlled superiority trial. Trials, 15, 112. https://doi.org/10.1186/1745-6215-15-112

3) In addition, the allocation status, which is at the center of the research, is a binary variable. Although sometimes binary variables are treated as independent variables, classically, dependent and independent variables should be numerical in regression analysis. In this case, it is confusing about the variable in the center of the research.

**Our response**: We agree with the reviewer that the dependent variable in the linear regression models used must be a metric variable. This requirement is met in all models in the manuscript. However, this requirement does not apply to independent variables. Binary variables can also be considered in regression models as independent variables in addition to metric variables. For example, in most regression models the binary variable "sex" is included as an independent variable. Hence, the inclusion of the variable "allocation status" as an independent variable in the linear regression models is correct from our point of view. Furthermore, the aim of the publication is "to examine variability in the course of anxiety symptom severity at 12 months follow up and to explore patient characteristics, which may explain that variability". Therefore, the objective is not only to show a possible correlation of the change in anxiety symptoms with the variable "allocation status", but also a possible correlation with other "patient characteristics”.
